# Bio-Hybrid Micro/Nanodevices Powered by Flagellar Motor: Challenges and Strategies

**DOI:** 10.3389/fbioe.2015.00100

**Published:** 2015-07-27

**Authors:** Jin-Woo Kim, Steve Tung

**Affiliations:** ^1^Bio/Nano Technology Laboratory, Institute for Nanoscience and Engineering, University of Arkansas, Fayetteville, AR, USA; ^2^Department of Biological and Agricultural Engineering, University of Arkansas, Fayetteville, AR, USA; ^3^Department of Mechanical Engineering, University of Arkansas, Fayetteville, AR, USA

**Keywords:** flagellar motors, bio-hybrid engineered systems, bio-MEMS, biosensing, medical diagnosis, nanobiotechnology

## Abstract

Molecular motors, which are precision engineered by nature, offer exciting possibilities for bio-hybrid engineered systems. They could enable real applications ranging from micro/nano fluidics, to biosensing, to medical diagnoses. This review describes the fundamental biological insights and fascinating potentials of these remarkable sensing and actuation machines, in particular, bacterial flagellar motors, as well as their engineering perspectives with regard to applications in bio-engineered hybrid systems.

## Introduction

Molecular motors are ubiquitous in biological systems and play vital roles in a wide variety of biological processes, including cell motility, organelle movement, virus transport, etc. (Schliwa, 2003). Examples of molecular motors include cytoskeletal motors, such as myosin, polymerization motors, such as actin, and rotary motors, such as F_1_-ATPase and bacterial flagellum. From an engineering point of view, these minute molecular machines offer exciting possibilities for a new generation of hybrid biomotor sensing and actuation systems with applications ranging from microfluidic mixers and motors, to chemical sensing, to medical diagnoses. They are the sensing and actuation systems precision engineered by nature over millions of years with unquestionable advantages over other chemo-mechanical systems, including no “wear-and-tear” (Schmidt and Montemagno, 2003), self-regulating and self-healing capabilities (Haimo, 2003), and very high [~100% (Oster and Wang, 2000)] energy conversion efficiency, to name a few. As a result, there has been a sizable interest in the development of molecular motor-based biosensing and bioactuation micro and nano systems (Soong et al., 2000, 2001; Schliwa, 2003; Schmidt and Montemagno, 2003; Soong and Montemagno, 2005; Xi et al., 2005; Al-Fandi et al., 2006; Kim and Tung, 2006; Tung and Kim, 2006, 2008; Wang et al., 2008; Tung et al., 2011). Molecular motors have been used to realize microfluidic actuators and sensors, including the nano-propeller system by attaching a nanofabricated nickel propeller to an F_1_-ATPase (Soong et al., 2000, 2001; Schmidt and Montemagno, 2003; Soong and Montemagno, 2005), and micro-electro-mechanical-system (MEMS)-based actuator (Al-Fandi et al., 2006; Tung and Kim, 2006, 2008; Tung et al., 2011), power generating devices (Tung and Kim, 2006, 2008), and sensor system (Kim and Tung, 2006; Tung and Kim, 2006, 2008) using bacterial flagellar motors. Despite recent progress, the field of engineering molecular motors for hybrid living-synthetic engineered systems is still in its infancy.

To realize the promising potential of molecular motors, the latest knowledge on molecular motors is essential not only for understanding various biological and biomedical topics but also for appreciating fascinating potentials of transforming the knowledge into the development of bio-engineered hybrid systems. The history and state of the art and science of molecular motors, in particular, F_1_-ATPase and flagellar motors, have been extensively reviewed elsewhere (MacNab, 1996; Boyer, 1997; Berry, 2003, 2005; Berg, 2003a,b; Schliwa, 2003; Sowa and Berry, 2008; Delalez, 2014). Among different types of molecular motors, this review focuses on bacterial flagellar motors and describes the fundamental biological insights of this remarkable sensing and actuation machine, as well as its engineering perspectives with regard to applications in hybrid living-synthetic engineered systems and nanobiotechnology. This review is not meant to be comprehensive. Here, instead, we attempt to stimulate discussions about practical strategies to realize the promise of bacterial flagellar motors for hybrid sensing and actuation systems. After a brief overview of the fundamental biological, chemical, and physical characteristics, such as the function and structure of bacterial flagellar motors, we present the current status and challenges, both biochemical and mechanical, in the design and integration of flagellar motors into micro- and nano-engineered systems, and discuss some biological and engineering strategies to overcome the challenges. Specifically, we consider the experimental realization of bio-inspired MEMS sensing and actuation systems powered by bacterial flagellar motors.

## Bacterial Flagellar Motor: Promises as Bioactuator and Biosensor

**Figure 1:**
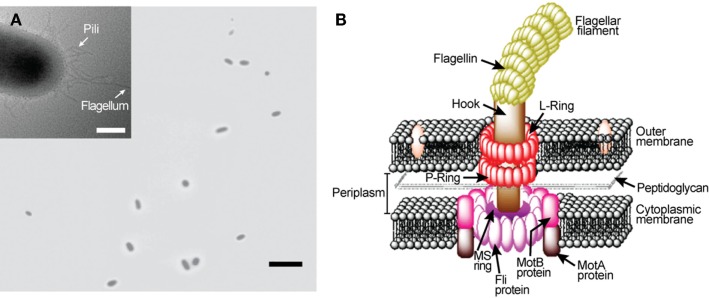
***Escherichia coli* rotary flagellar motor**. **(A)** Light microscopy image of free-swimming *E. coli* cells. Inset: transmission electron microscopy image of an *E. coli*. **(B)** Schematic of *E. coli* rotary flagellar motor.

Numerous bacteria species swim around to find favorable conditions for their survival. Flagellar motors (Figure 1), found in many bacterial species including *Escherichia coli* and *Salmonella enterica typhimurium*, are precision engineered by nature to provide cellular locomotion in response to environmental stimuli, i.e., tactic response (MacNab, 1996). Motor control is regulated through mechanisms that both sense the environment and communicate sensed information to the motor output to actuate the motor’s rotation. Figure 1B displays the primary structural components of a flagellar motor. A motile *E. coli* cell typically has four to eight flagellar motors embedded in the cell envelope at random points on the cell body (Figure 1A) (Manson, 2010). Each motor, 45 nm in diameter, is constructed from about 20 different proteins that work in tandem and function like a man-made stepping motor (Figure 1B). The L and P rings are the bushing for the driveshaft (rod). The MS and C rings constitute the rotor, which is surrounded by a ring of about 11 stator particles embedded in the cytoplasmic membrane. Each flagellar motor is connected to a helical flagellar filament through a universal joint called the hook. Through this structure, the motor spins the filament at a very high speed of over 100 cycles per second. The four rotating filaments on the bacterial cell body allow the cell to swim either in search of nutrients or away from repellants. Each flagellar filament, formed by subunits of the protein flagellin, is about 10 μm long with a diameter of about 20 nm. A flagellar motor can produce a power output of 10^−15^ W (Berry, 2003). Also, it can produce a torque level of 4 nN–nm (Lowe et al., 1987; Berry and Berg, 1997). The flagellar filaments and their cell bodies exist in an environment of very low Reynolds number where the viscous force dictates the fluid flow (Purcell, 1977).

**Figure 2:**
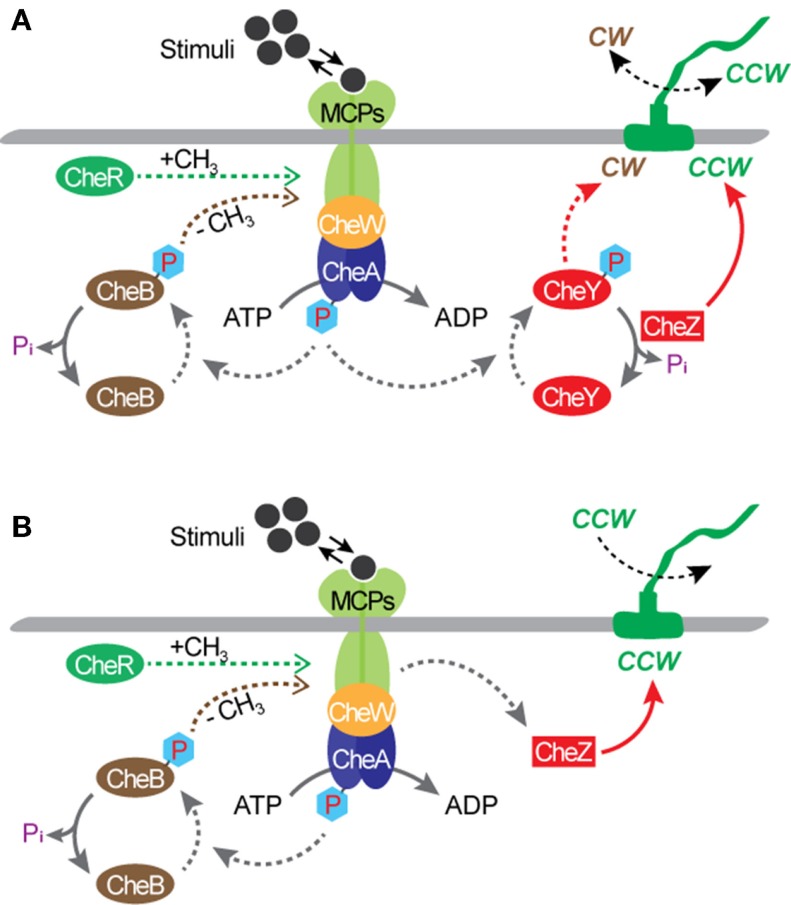
**Chemotactic circuitry**. **(A)** A wild type *E. coli*. **(B)** A mutant strain with a deletion of the *cheY* gene, such as *E. coli* KAF95 (Turner et al., 2000). With this mutation, flagellar filaments turn exclusively in the CCW direction.

Bacterial flagellar motors are powered by protons, rather than adenosine triphosphate (ATP) required by most molecular motors, such as an F_1_-ATPase motor (MacNab, 1996; Boyer, 1997). The transfer of protons from the environment to the cell is driven by an electrochemical gradient, maintained by the cell’s metabolism, across the cytoplasmic membrane. The motor of a wild type *E. coli* cell can rotate in both directions and a great deal is known about the molecular biology and biophysics of the rotational mechanism (MacNab, 1996; Berry, 2003, 2005; Berg, 2003a,b; Sowa and Berry, 2008; Manson, 2010; Delalez, 2014). When the motor rotates in the counterclockwise (CCW) direction, the filaments form a tight bundle and the bacterial cell “runs” or swims forward. When it rotates in the clockwise (CW) direction, the filaments move away from one another and the cell “tumbles” and swims in a new direction. Normally, each flagellar filament turns in the CCW direction about 80% of the time. The rotational directions are modulated by the environmental chemical stimuli through a process known as chemotaxis. The mechanism of chemotaxis involves a sequence of complex biochemical processes that require the cell to be equipped with a means of sensing the environment and a means of communicating the sensed information to the motor output. In *E. coli*, sensing is achieved by cell membrane-bound chemo-receptors called as the methyl-accepting chemotaxis proteins (MCPs). Signals from the MCPs control the direction of flagellar rotation via the sensor kinase CheA and regulator protein CheY. According to the current model (Figure 2A), when MCP binds to a chemical, it changes the autophosphorylation rate of CheA, yielding CheA-P. Attractants decrease the rate of autophosphorylation, whereas repellants increase the rate. When the rate is low, the rotational direction is CCW. When the rate is high, CheA-P phosphorylates CheY, forming CheY-P, and CheY-P induces CW rotation of the motor. Another regulator protein, CheZ, is responsible for dephosphorylating CheY-P, allowing the motor to recover from CW rotation and resumes CCW rotation. Also, motor rotation can be affected by the degree of MCPs’ methylation, which is regulated by another response regulator CheB and a cytoplasmic protein CheR. The CheB-P, phosphorylated by CheA-P, removes methyl groups from MCPs and the CheR continuously methylates MCPs at a slow rate. In the presence of attractants, the CheA-P level is low and therefore CheB-P as well as CheY-P are low. As a result, the level of MCPs methylation increases until fully methylated. Once fully methylated, MCPs do not respond to attractants anymore, resulting in an increase in the levels of CheA-P, CheB-P, and CheY-P. When this occurs, the cell tumbles. Simultaneously, the CheB-P demethylates MCPs, making them responsive to attractants again. In the presence of repellants, the opposite mechanism takes place; thus, the MCPs’ response to repellants becomes stronger as their methylation level increases. This implies that the sensitivity of the flagellar motor’s response to environmental stimuli could be controlled by modulating the level of MCPs methylation. Chemotaxis is controlled by gene products, i.e., proteins, and not by their synthesis. This is different from other two-component signal transduction systems, such as the regulation system of the nitrate reductase gene pathway. As a result, the speed of chemotactic response is much higher than that of other regulatory systems that require regulations at the gene expression level. For *E. coli*, directional switching was shown to occur within 1 ms in a single filament (Kudo et al., 1990). This characteristic suggests the great potential of flagellar motors as a rapid bio/chemical sensor for repellants that can induce a directional change in the motor rotation.

**Figure 3:**
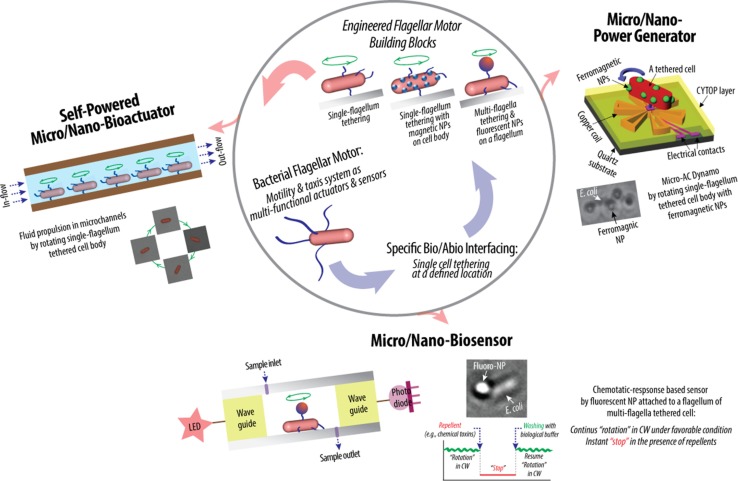
**Schematic of hybrid synthetic-living systems for multi-functional sensing, actuation, and power-generation using bacterial flagellar motor building blocks that interface with micro/nano-fabricated system surfaces in controlled manners** (Al-Fandi et al., 2006; Kim and Tung, 2006; Tung and Kim, 2006, 2008; Wang et al., 2008; Tung et al., 2011).

Much is known about bacterial genomes, including *E. coli*, and its regulation of the cell function. Based on this knowledge, genetic engineering has been applied to create mutant cells to control the rotational behavior of flagellar motors. Incidentally, some of these mutations also create opportunities for transforming microbial cells from free-swimming cells into micro engineering devices. A mutant strain of *E. coli* KAF95 is one such example (Figure 2B). The KAF95 cells, originally developed for studying the biophysical properties of the flagellar motor, carry two mutations (Turner et al., 2000). The first mutation is a deletion of the *cheY* gene, which regulates the motor’s CW rotation. Without this gene, the flagellar motor rotates exclusively in the CCW direction. The second mutation is an internal deletion of the *fliC* gene encoding the protein flagellin, which leads to a non-specific binding interaction between the flagellar filament and various substrate materials including glass and some polymers (Turner et al., 2000). With these mutations, a single, shortened filament of a motile KAF95 cell can be tethered down to a flat substrate with the motor turning the cell body spontaneously in the CCW direction at about 10 Hz. In this configuration, the rotating cell body, about 3 μm long and 1 μm in diameter, can be considered as a micro-actuator in a microfluidic system. This suggests the excellent possibility of *E. coli* flagellar motors as both actuators and sensors integrated into actual engineering devices, developing hybrid synthetic-living systems for multi-functional sensors and actuators, including microfluidic valves, mixers, and motors (Figure 3).

## Flagellar Motors for Hybrid Micro/Nanodevices: Current Status and Challenges

Highly efficient sensing and actuation as well as tethering capability of genetically engineered microbial cells present their potential for bio–abio integrated MEMS sensing and actuation devices to converge onto successful integration of living and engineered systems. This might make real applications in biosensing, micro/nano fluidics, etc., producing novel micro/nano devices for drug delivery, energy conversion, real-time detection of chemical and biological toxins, etc. (Figure 3). Specifically, the high power and high torque capability of flagellar motors suggest the possibility of using flagellar motors in a hybrid system for micro-actuation as well as micro power generation. Furthermore, the chemotactic responses of flagellar motors toward repellents, such as nitrate and nitrite, suggest the possibility of using flagellar motors in a hybrid system for the detection of harmful chemicals, such as nitroaromatic and organic nitrate explosives. In flagellar motor research, the majority of scientific studies were focused on uncovering the biochemical and biophysical properties of the motors and their tactic responses (Lowe et al., 1987; Kudo et al., 1990; MacNab, 1996; Berry and Berg, 1997; Turner et al., 2000; Berry, 2003, 2005; Berg, 2003a,b; Sowa and Berry, 2008; Manson, 2010; Delalez, 2014). In contrast, the engineering techniques required to interface and integrate flagellar motors into actual engineering devices remain a significant challenge and largely under explored. The challenges are to create a micro/nano system that has a predetermined number of flagellar motors at designed locations and orientation, to sustain the motors’ activity and optimize their sensitivity, which are tailored to specific applications, and to design and fabricate micro/nano systems with integrated modules for sensing and control. All of these requirements contribute to the establishment of control of the system integration process in order for the designed flagellar motor-based device to be constructed with minimum unwanted products and errors.

### Specific bio–abio interfacing

A prerequisite to the integration of bacterial flagellar motors with micro/nano-fabricated devices is a controlled interfacing of biological, i.e., bacterial cells, and abiological, i.e., MEMS or nanoparticles, components at similar scales, allowing control over the number and location of cells in the MEMS system. The micro/nano systems with control over cell number and location would enable enhanced control over their functions. This would also make the production process of the hybrid systems reproducible, allowing their mass-production with well-defined functions. However, such controlled interfacing is not trivial and not only should the interplay of biophysicochemical and mechanical properties of both biological and abiological components be considered but careful rational design is also required for the development of the bio-hybrid micro/nano systems with desired functions. These include three parts that dynamically interact each other: (1) the abiological surface, e.g., MEMS channel surface, the properties of which depend upon its physicochemical characteristics, including charge, roughness, and accessible surface area as well as available functional groups and ligands, (2) the biological component, i.e., bacterial flagella, the properties of which rely upon its biophysicochemical characteristics, including length, charge, hydrophilicity and hydrophobicity, and available functional groups and ligands, and (3) the bio–abio interface where the biological component contacts with the abiological via one or combinations of electrostatic, electrosteric, steric, hydrophilic and hydrophobic, and ligand–receptor binding interactions.

**Figure 4:**
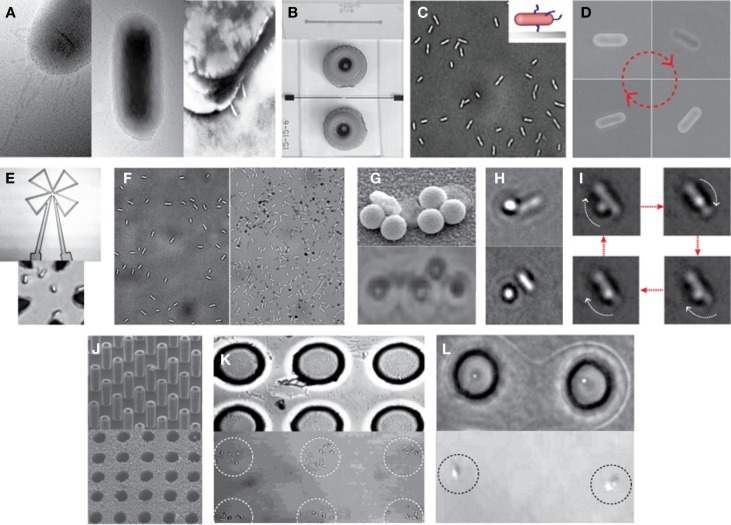
**Examples of hybrid micro-systems powered by bacterial flagellar motors**. **(A)** Shearing flagellar filaments: transmission electron microscopy (TEM) images of an *E. coli* cell before (left) and after (middle and right) the shearing process according to Al-Fandi et al. (2006). Right image represents a cell image after washing the sample (middle) at the end of the shearing process (Wang et al., 2008). **(B)** Typical microfluidic systems: scanning electron microscopy (SEM) image of microchannel formed in PDMS (top) and light microscopy (LM) image of a top view of a completed microfluidic system with microfluidic connectors (bottom) (Tung and Kim, 2008). **(C)** Single-flagellum tethering: LM image of *E. coli* cells tethered to a glass substrate through a short flagellar filament. Inset: schematic illustration of a tethered cell with shortened flagella. **(D)** LM image of a rotational sequence of an *E. coli* cell tethered through a short filament (Al-Fandi et al., 2006). **(E)** Cell integration with a microcoil chip for power generation: LM image of microcoil (top) and *E. coli* cells with short flagellar filaments tethered at the center of the microcoil (bottom). **(F)** Effect of micro/nano-surface texturing on cell adhesion: LM images of *E. coli* cells with short filaments on a smooth glass surface (left) and micro/nano-textured surface (right) prepared by AIC of amorphous silicon with an average texture size of about 600 nm (Wang et al., 2008). **(G)** A single-flagellum tethered *E. coli* with multiple particles: TEM image with 0.5-μm polystyrene beads (top) (Tung and Kim, 2006) and LM image with 0.2-μm ferromagnetic NPs (bottom). **(H,I)** Multi-flagella tethered *E. coli* with a bead on the tip of a rotating flagellar filament: LM images of *E. coli* with a 0.5-μm fluoro-bead on a shortened flagellum [**(H)**, top] and on a relatively long (i.e., moderately shortened) flagellum [**(H)**, bottom] (Tung and Kim, 2008), and the rotational sequence of the bead **(I)**. **(J)** Microfabricated sieve: SEM images of micro-silicon master posts (top) and a finished PDMS micro-sieve (bottom) (Tung and Kim, 2008). **(K)** Micro-sieve technique to control the location of tethered cells: *E coli* tethered in 10-μm holes of micro-sieve (top) and those after peeling of the micro-sieve (bottom) (Tung et al., 2011). **(L)** Combination of dip-pen technique with micro-sieve: LM images of the single-cell pattern generated before (top) and after (bottom) peeling of the micro-sieve (Tung et al., 2011). Figures were adapted with permission from [**(A)** (right), **(B,F,G)** (top), **(H,J)**] Wang et al. (2008), Tung and Kim (2008), Wang et al. (2008), Tung and Kim (2006), Tung and Kim (2008), and Tung and Kim (2008), respectively, copyright 2008, 2008, 2008, 2006, 2008, and 2008, respectively, IEEE; **(D)** Al-Fandi et al. (2006), copyright 2006 Elsevier; **(K,L)** Tung et al. (2011), copyright 2011, Wiley.

Recently, Kim and Tung reported a series of hybrid micro-systems powered by bacterial flagellar motors (Al-Fandi et al., 2006; Kim and Tung, 2006; Tung and Kim, 2006, 2008; Wang et al., 2008; Tung et al., 2011). These include flagellar motor-based self-powered micro-actuator, AC dynamo, and biosensor systems (Figure 3). In these studies, they demonstrated the tethering of *E. coli* KAF95 cells to various surfaces, including glass and metals, by a single or multiple flagellar filament(s) depending upon the filament’s length without any special pretreatments (Figure 4). Single-flagellum tethering was realized when the filament was shortened to about 0.5 μm from the original 10 μm (Figure 4A) for micro-actuation (Figures 4B,C) and power-generation systems (Figure 4E), where the cell body turns like a merry-go-around in the CCW direction at 10 Hz (Figure 4D) (Al-Fandi et al., 2006; Tung and Kim, 2006, 2008; Tung et al., 2011). Shortening the filaments was shown to substantially increase the rotational efficiency of the tethered cells, indicating increased probability of cell tethering by a single flagellum. Multi-flagella tethering was achieved when the filament was relatively long (>2 μm) for the biosensing system (Kim and Tung, 2006; Tung and Kim, 2006, 2008), where the cell body sticks to the surface and the filament on top of the tethered cell rotates freely at a high rotation rate, i.e., 100 Hz (Figures 4H,I). Also, they successfully demonstrated the attachment of a single micro/nano particle, including 0.5 μm polystyrene beads (Figure 4G, top), 0.2 μm ferromagnetic particles (Figure 4G, bottom), and a 0.5-μm fluoro-bead (Figures 4H,I) on the tip of a single “sticky” flagellar filament. Nano-surface-texturing was used to further promote cell-to-surface adhesion and control the distribution of tethered cells (Wang et al., 2008). Using nano-surface-texturing prepared by aluminum (Al)-induced crystallization (AIC) of amorphous silicon with an average texture size of about 600 nm, the tethering efficiency was increased by as much as 300% (Figure 4F). However, filament tethering was a very tedious and trial-and-error-based process due to the need for a delicate shearing procedure to obtain the desired filament lengths. It included shearing and shortening the flagellar filaments by passing the cell cultures back-and-forth between two polyethylene tubes (Figure 4A) (Tung et al., 2011). Moreover, preferential locational control of cell tethering was very difficult to achieve due to the “sticky” nature of the flagella of the KAF95 cells. They bind to all materials tested regardless of charge properties and hydrophobicity of the materials (Tung and Kim, 2006, 2008), suggesting that their binding is non-specific in nature. To realize flagellar motor-based micro/nano-fabricated devices, the attachment of cells to the surface should be very selective and specific. A clever approach was attempted to achieve specific tethering by the use of a microfabricated sieve and dip-pen technology (Figures 4J–L) (Tung et al., 2011). A flexible PDMS membrane with arrays of patterned holes was fabricated on a microfluidic channel (Figure 4J). The micro-sieve technology alone could control the locations of tethered cells (Figure 4K) and the number of tethered cells by controlling the sieve hole size and cell concentrations in the sample. However, single cell tethering was very difficult to achieve with the micro-sieve alone because as the cell concentration and sieve hole size were reduced, the probability for a single cell to enter a sieve hole was also reduced. To overcome the technical challenges for single-cell tethering, cells were selectively deposited in the sieve holes by utilizing a dip-pen (Figure 4L, top), followed by peeling off the micro-sieve after cell tethering (Figure 4L, bottom). The microfabricated sieve along with the dip-pen technology allowed single cell tethering at a specified location, representing the first patterning technique to successfully tether single *E. coli* bacterial cells via their flagellar filament. However, this method was not always consistent due to the fact that the user cannot precisely control the number of cells during the dip-pen procedure. To achieve true single cell patterning at defined locations using the dip-pen technology, a single cell must be present per deposition at each sieve hole, which is hard to achieve with the existing technology. Hence, great demand still exists for controlling the placement of cells at designed locations by controlling their interactions with the microfabricated surface, which is imperative to engineer flagellar motor-based hybrid sensing and actuation systems. This will require synergistic efforts involving interdisciplinary research of diverse areas, including molecular biology, interfacing technology, and micro/nano system engineering. This may include design strategies to genetically engineer flagellar filaments that will provide not only specific and robust attachment of flagellar filaments on the microchannel by introducing affinity tags, such as His- or biotin-tag, into flagellin but also pre-defined length of flagella by careful deletions of internal flagellin proteins. Also, an advanced 2D or 3D micro/nanoscale surface patterning would be necessary that enables site-directed attachment of cells with control over their placement. A technology to be considered to modulate cell attachments includes the self-assembled 2D and 3D nanoscale patterns (Kim et al., 2011, 2012; Kim and Deaton, 2013) that can serve as 2D and 3D arrays for attaching cells to the designated locations of the pattern via a specific ligand–receptor binding interaction.

### System sensitivity and reliability

In addition to specific cell tethering, an equally important challenge in a hybrid system is to optimize and maintain the cell activity, such as flagellar motor rotation, as well as to achieve specificity required for its sensing application. Maintaining activities of cells is critical for reliable performance of the system. A previous study showed that a tethered *E. coli* rotates continuously for an average of 24 h in a microchannel filled with a standard tethering buffer solution (sodium phosphate buffer with EDTA, pH 7). To maximize the life span of a tethered cell and its rotational efficiency, the system would require the incorporation of a nutrient feeding mechanism. This mechanism must be carefully designed to avoid either over or under feeding. Overfeeding can result in undesirable cell growth and division, which will affect the engineering performance of the hybrid sensing system. Candidates for such nutrient additives include a metabolizable carbon source, such as d-lactate, and a final form of energy source, such as ATP. A controlled nutrient release system could be incorporated into the hybrid MEMS system to supply nutrients on demand or through maintenance of nutrient levels within a desired range for cells to be stable and consistently active for a longer period of time. “Intelligent” stimuli sensitive polymers with the unique capability to change structure in response to environmental changes, such as temperature, pH, and electrolyte, including a poly-acrylic acid (PAA) that is sensitive to pH and poly-*N*-isopropylacrylamide (PNIPAAm) that is sensitive to temperature (Brannon-Peppas, 1997; Liechty et al., 2010), would allow us to design a controlled nutrient release system, through which nutrients can be supplied when needed. For example, the nutrient release system, if temperature sensitive polymers are used, starts heating when changes in cell rotation occur, supplying nutrients to the cells to recover the cell’s rotational activity.

While “bio-inspiration” has motivated research in sensor developments, it is rare for a bio-engineered system to be developed to meet all the requirements of a practical sensor: fast response time, sensitivity, specificity, stability, and portability. With existing detection technologies, increased system response time and sensitivity requirements translate directly into increased system size, weight, power requirements, and cost. Most of the current detection systems have significant support requirements due to the use of wet chemistry and expensive and sensitive agents. The use of expensive and sensitive reagents is a huge logistics burden on the user. Thus, a challenge is to discover ways to detect a great variety of toxic agents in environmental samples in real time, and with high sensitivity and low cost, and to warn the spread of contamination, preventing exposure. A prior study demonstrated that, using nitrite as a model repellant, a flagellar motor sensing system has the limit of detection (LOD) of ~300 μM, which is comparable to the existing biosensors with the LODs ranging from ~0.1 to 700 μM (Almeida et al., 2010; Yilong et al., 2015), and that the response time of the flagellar motor biosensor is almost instantaneous due to the fast chemotactic response of flagellar motors to external stimuli (Figure 3), which is difficult to achieve with existing technologies. Although this study was highly promising, another key challenge to develop signal-specific sensing systems identified is the lack of specificity in chemotactic responses of bacterial cells. Biological and system-level optimization of the sensing and actuation properties of flagellar motors, in particular, the chemotactic specificity and sensitivity to specific target toxins, are required. There would be room for improving the sensitivity as well as the speed of detection by optimizing the level or physiological status of the components used in sensing and actuation of the flagellar motor, considering that chemotaxis is regulated by the activity of existing proteins in the cell. Also, by close examination of current models for chemotaxis, intracellular steps or processes critical to directing the chemotactic response may also be altered by mutagenesis in order to achieve increased signal sensitivity and specificity. On the other hand, real-time detection of chemical and biological toxins in the environment is challenging because of the number of potential agents to be distinguished, the complex nature of the agents themselves, and the myriad of similar agents that are a constant presence in the environment. Thus, chemotactic systems capable of non-specific identification, e.g., determining the presence of chemical and biological toxins by targeting generic factors, could be highly desirable in some applications. Although much still needs to be done, results like this serves as an important step toward the development of future hybrid systems of rotational cells and micromachined components.

## Summary and Future Prospective

This review has highlighted the design and realization of bio-inspired sensing and actuation of micro/nano-engineered systems powered by molecular motors, in particular, bacterial flagellar motors. Key challenges for integration of flagellar motor function into hybrid sensing/actuation systems were outlined and potential routes to overcome the challenges were suggested. Emphasis was put on the necessity of control to achieve desired interfacing between the flagellar motor and micro/nano-fabricated system, as well as the motor’s desired function, i.e., sensitivity and specificity, tailored to specific applications and its reliability. We addressed the progress and promise of experimental realization of their interfacing with control over the number and location of and distance between flagellar motors in a MEMS system, and proposed efficient and practical methods, both biological and engineering strategies, to not only achieve specific bio–abio interfacing but also optimize and prolong a motor’s function. However, further research on fundamental biology and engineering to better understand the sensing, actuation, and dynamic control processes is required in order to fully take advantage of the superior biological system that nature has evolved over millions of years. A better understanding of biological and engineering solutions would allow us to overcome challenging system-level problems and significantly improve their performance. This would provide a firm foundation for rapid technical innovation to develop practical flagellar motor-based hybrid multi-functional sensors and actuators, with a broad range of applications that impact the nation’s economy, security, and the quality of life.

## Conflict of Interest Statement

The authors declare that the research was conducted in the absence of any commercial or financial relationships that could be construed as a potential conflict of interest.
